# Patient experiences during the COVID-19 pandemic: a qualitative study in Dutch primary care

**DOI:** 10.3399/BJGPO.2022.0038

**Published:** 2022-11-30

**Authors:** Maarten Homburg, Daan Brandenbarg, Tim olde Hartman, Lotte Ramerman, Gina Beugel, Corinne Rijpkema, Robert Verheij, Marjolein Berger, Lilian Peters

**Affiliations:** 1 Department of General Practice and Elderly Care Medicine, University of Groningen, University Medical Center Groningen (UMCG), Groningen, The Netherlands; 2 Department of Primary and Community Care, Radboud University Nijmegen Medical Center, Radboud institute of Health Sciences, Nijmegen, The Netherlands; 3 Nivel, Netherlands Institute for Health Services Research (Nivel), Utrecht, The Netherlands; 4 Tilburg School of Social and Behavioral Sciences, Tilburg University, Tilburg, The Netherlands; 5 Amsterdam University Medical Center, Vrije Universiteit Amsterdam, Midwifery Science, AVAG, Amsterdam Public Health, Amsterdam, The Netherlands

**Keywords:** continuity of patient care, health services accessibility, primary health care, COVID-19, healthcare utilisation

## Abstract

**Background:**

Changes in primary care provision during the COVID-19 pandemic could have affected patient experience of primary care both positively and negatively.

**Aim:**

To assess the experiences of patients in primary care during the COVID-19 pandemic.

**Design & setting:**

A qualitative study of patients from regions with high and low COVID-19 prevalence in the Netherlands.

**Method:**

A qualitative study using a phenomenological framework was performed among purposively sampled patients. Individual semi-structured interviews were performed and transcribed. Data were thematically analysed by means of an inductive approach.

**Results:**

Twenty-eight patients were interviewed (13 men and 15 women, aged 27–91 years). After thematic analysis, two main themes emerged: accessibility and continuity of primary care. Changes considered positive during the pandemic regarding accessibility and continuity of primary care included having a quieter practice, having more time for consultations, and the use of remote care for problems with low complexity. However, patients also experienced decreases in both care accessibility and continuity, such as feeling unwelcome, the GP postponing chronic care, seeing unfamiliar doctors, and care being segregated.

**Conclusion:**

Despite bringing several benefits, patients indicated that the changes to primary care provision during the COVID-19 pandemic could have threatened care accessibility and continuity, which are core values of primary care. These insights can guide primary care provision not only in this and future pandemics, but also when implementing permanent changes to care provision in primary care.

## How this fits in

Changes in primary care provision during the COVID-19 pandemic have affected accessibility and continuity of primary care for patients. The study showed that patient experiences during the COVID-19 pandemic mainly concerned accessibility and continuity, which are core values of primary care. This study provides insights into barriers and opportunities in primary care as experienced by patients, which could be used for optimising primary care during and after the COVID-19 pandemic.

## Introduction

The COVID-19 pandemic had an immense impact on the organisation and utilisation of primary care.^1–3^ General practices were forced to transform their model of care to cope with not only the sudden and significant burden of COVID-19 and related care, but also to minimise the risk of COVID-19 transmission in healthcare settings.^4–9^ For example, practices often substituted face-to-face consultations for telephone consultations and other digital consultation methods.^5^ These changes may have affected how patients experienced both care accessibility and the continuity of care by their GP.^10^

The widely accepted core values of primary care, that it should provide easily accessible, continuous, comprehensive, and coordinated care,^11,12^ are associated with improved population health.^11,13^ This particularly applies to healthcare systems in which GPs act as gatekeepers to secondary care, such as The Netherlands..^14^ This type of system can lower healthcare use and improve care quality,^14^ but health outcomes can also be negatively affected when accessibility and continuity of care are impaired.^11,13^ The COVID-19 pandemic exacerbated precisely this situation owing to the necessary restrictive measures and organisational changes. For example, patients avoided GP care for different reasons, such as fear of becoming infected with COVID-19 or of being a burden on the healthcare system.^7^ Describing patient experiences during the pandemic is valuable since it can provide understanding regarding what is important in primary care according to patients. These insights could be used for optimising primary care.

This study assess the experiences of patients with and without COVID-19 regarding primary care during the COVID-19 pandemic.

## Method

### Design

This qualitative study used a phenomenological framework to explore patient experiences regarding healthcare provision in primary care during the COVID-19 pandemic.^15^ The purpose of this approach is to describe and understand the lived experiences with a phenomenon. Semi-structured interviews were conducted to gain insights into the positive and negative experiences of patients regarding primary care, focusing on the GP, during the COVID-19 pandemic in The Netherlands.. The study is reported according to the COREQ (COnsolidated criteria for REporting Qualitative research) statement.^16^

### Study population and recruitment

Patients were eligible for participation if they required care from their GP between March 2020 and May 2021, irrespective of the reason for consultation. Furthermore, patients had to be adults to participate, and had to be able to participate in an interview. During the inclusion period, restrictive measures varied between strict (lockdown) measures and less strict measures in periods between pandemic waves. Recruitment occurred via the following three sources: (1) a Dutch patient association, Zorgbelang; (2) a Dutch organisation for empowering people who live in poverty and have experienced social exclusion (Strong from Poverty Foundation; in Dutch, Sterk uit Armoede); and (3) GPs associated with the study and from personal networks. Purposive sampling was then used to ensure variation in age (young, middle-aged, and older adults), sex, self-reported COVID-19 infection (yes or no*),* comorbidity, educational level, and region. A maximum variation sampling framework was used. During the pandemic, the northern region of The Netherlands. initially had a relatively low prevalence of COVID-19, whereas the southern region had a high prevalence; the middle region had an average prevalence compared with these.^17^ Patients were selected for participation by the research team based on the desired characteristics of the purposive sample. Potential participants received background information about the study aim, emphasising that all identifiable data were removed from the interview transcripts; that participation was voluntary; and that they could withdraw from the study at any time. Informed consent was obtained from each participant before interview. Participants received an incentive worth €25 for participation. After the data analysis, an overview of the study results was fed back to the participants.

### Data collection

The semi-structured interviews were held by telephone or video-conferencing, discussing topics on a topic list developed to answer the research question (Supplementary Box S1). Consensus regarding the topic list was reached within a research team comprising researchers and GPs. Furthermore, a pilot version of the topic list was discussed with patient representatives and a GP advisory board that included 10 GPs associated with the University Medical Centre Groningen, Radboud University Nijmegen, and Maastricht University Medical Centre. The main question of interest was ‘Did you notice any changes in primary care after the start of the pandemic and how did you experience them?’. Three trained interviewers (CR, WB, DB) conducted the interviews and were free to adapt questions within these topics according to the situation. The interviewers and participants had no personal relationships. If new topics arose from the interviews, they were added to the topic list and used in subsequent interviews. When data saturation was suspected, five additional interviews were held to check if they yielded additional information. If no additional information was obtained after these five interviews, data saturation was assumed and no additional interviews were held. All interviews were audio-recorded and complemented by field notes after receiving permission from participants. During the interviews, patients were regularly asked if the obtained information was interpreted correctly. After completion of the interview, the participants were asked if they had any additional comments, if certain topics needed more attention and if specific topics were lacking. Field notes were discussed with the participant for verification of the obtained data. The recordings were then transcribed verbatim and pseudonymised.

### Data analysis

Data collection and analysis occurred iteratively, with data management and coding performed using ATLAS.ti (versions 8.4 and 9). Transcribed interviews were analysed using an inductive thematic approach and data was coded open, axial, and selective.^18^ Two separate researchers (GB, CR) independently marked relevant transcript segments related to the research question and assigned codes. Independently coded interviews were discussed between two researchers and new codes were assigned based on consensus. If consensus was not achieved, these segments were discussed with a third researcher (DB) who made the final decision. After data collection, the core team (MH, GB, DB) discussed the codes to identify potential patterns in the data and defined themes and sub-themes.

## Results

### Participants

Interviews were held with 13 men and 15 women (aged 27–91 years; nine had previous COVID-19 infection) between January 2021 and June 2021 (Table 1). Saturation regarding patient experience was suspected after 23 interviews and no additional themes emerged from the five subsequent interviews. No patient withdrew their consent. No new topics arose during the interviews.

**Table 1.** Characteristics of the interviewed patients (*n* = 28)

ID	Sex	Age, years	Education	Region	COVID-19	Contact^a^	OOH	Chronic condition
1	Male	66	Middle	North	No	T, C	No	Oncological
2	Female	75	High	North	No	T	No	Diabetes
3	Female	38	Middle	South	No	T, C	No	Gastrointestinal
4	Male	50	High	South	No	C	Yes	Mental health
5	Female	57	Middle	North	No	T, C	No	Oncological
6	Male	51	Low	South	No	C	No	Diabetes, COPD, cardiovascular
7	Male	71	High	North	No	C	No	None
8	Male	91	High	South	No	C, V	No	Oncological
9	Female	55	Middle	South	No	T, C	No	COPD, cardiovascular
10	Male	57	High	North	No	None	No	None
11	Female	59	Low	South	No	C	No	Gastrointestinal
12	Male	81	High	North	No	T	No	Diabetes
13	Female	55	High	South	No	T, C	No	COPD, musculoskeletal
14	Female	38	Middle	North	No	T, C	No	Musculoskeletal
15	Female	33	High	North	No	T, C	Yes	Mental health
16	Female	52	Middle	North	No	T, C	No	Musculoskeletal
17	Female	55	High	North	Yes	T, C	Yes	COPD
18	Female	45	Middle	North	Yes	T, C	No	None
19	Male	65	Middle	North	Yes	T, C, V	Yes	None
20	Male	45	High	Middle	Yes	T, C, V, CI	No	COPD
21	Male	72	Low	North	No	T, C	No	None
22	Female	36	High	Middle	Yes	T, C	Yes	None
23	Female	68	Middle	North	No	T, C	Yes	Cardiovascular
24	Female	64	Middle	North	No	T	No	None
25	Male	59	High	North	Yes	T, V, CI	Yes	None
26	Woman	27	High	South	Yes	T	No	None
27	Male	60	Low	Middle	Yes	T, C	Yes	COPD, cardiovascular
28	Male	51	High	Middle	Yes	T, C,	Yes	None

^a^Type of contact: T = telephone; C = regular consultation; V = visit at home; CI = consultation at specific infection location. COPD = chronic obstructive pulmonary disease. OOH = out of hours.

### Thematic analysis

**Figure 1.**
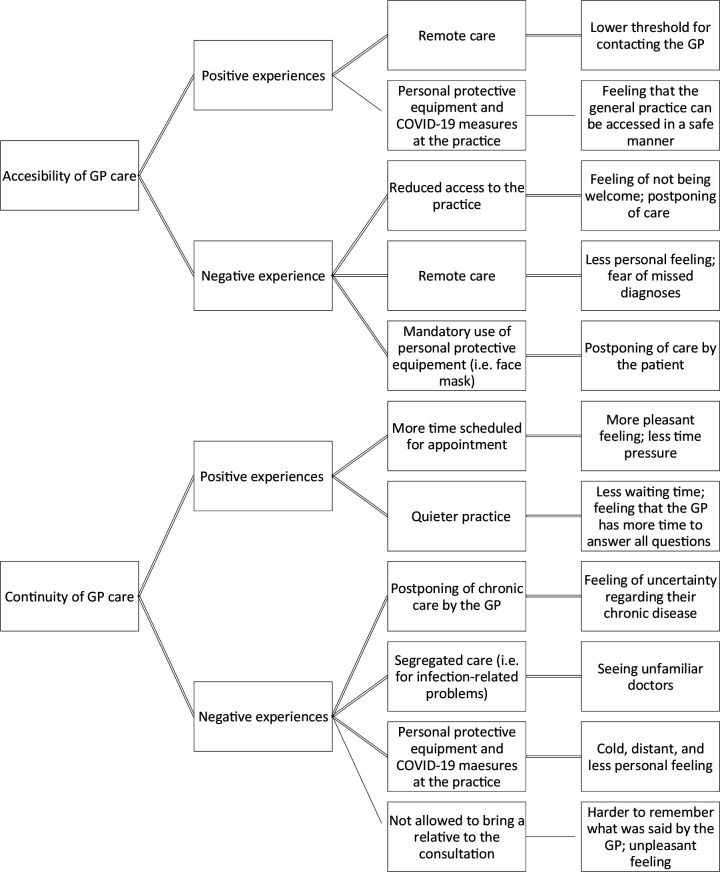
Subdivision of patient experiences in positive and negative experiences, and subsequent consequences according to the patients

Primary care accessibility and continuity were identified as the main themes. Patients experienced positive and negative changes in both primary care accessibility and continuity. Therefore, patient experiences of care were organised within the overarching themes of accessibility and continuity, sub-themes of positive and negative experiences, and sub-sub-themes of consequences (Figure 1).

#### Care accessibility

Access to general practice was often not allowed without an appointment during the COVID-19 pandemic. This led some patients to feel unwelcome at the practice, which increased their threshold for making appointments when they needed care. They stated that this had caused them to postpone or avoid care.

*'You really have the feeling that you have to fight for every piece of care you want to receive, and even then, it was held off. It feels like you’re being kept away.'* (ID17)

Patients had both positive and negative experiences of the increased use of remote care, including email contact, video-calling, and other e-health applications. For low complexity problems, they received adequate care without needing to visit the practice. Remote care, therefore, proved to be a time-saving and practical approach that lowered the threshold for contacting the GP:

*'I think it* [remote care] *is a great way to have a low threshold for being in contact with your GP.'* (ID10)

Patients typically had negative experiences of remote care when used for more complex problems, experiencing it as distant and less personal. The interviews revealed the importance of looking the GP in the eyes and of receiving a physical examination. When absent, these led to feelings of reduced care quality and increased stress. Some patients also stated that telephone contact made it harder to remember what they discussed:

*'When I am at the general practice and I am not feeling well, the doctor can easily listen to my heart and lungs. This is not possible in that situation* [remote care]*. Maybe the GP makes* [wrong] *decisions based on remote care alone.'* (ID27)

Measures taken to prevent the spread of COVID-19 in general practice, such as distance measures and personal protective equipment, also led to positive and negative experiences. The measures reassured some patients that they could visit the practice safely with minimum risk of becoming infected:

*'Given the circumstances, it* [COVID-19 measures at the general practice] *is very positive …* [and] *… gives the feeling that you can visit the GP in a safe manner. Personally, I think it is very good that it is done this way.'* (ID9)

By contrast, other patients stated that these measures had led to a feeling of more impersonal care, describing the general practice as cold and distant. This had the potential to increase the threshold for visiting the general practice. One of the patients underlined this by saying:

*'When you enter the waiting room and you see taped squares on the ground and you’re sitting in an empty space without a reading table, it feels cold and clinical. It is a practice … it doesn’t need to be a living room, but right now it is really clinical and, I think, impersonal.'* (ID22)

#### Continuity of care

Multiple patients reported appreciating that they had more time scheduled for GP appointments during the COVID-19 pandemic, which they thought had improved the quality of care. In their opinion, this led to a more pleasant and relaxed situation because they had more time to talk with the GP and felt less time pressure. Patients experienced practices as being quieter with few patients around and, given that this reduced waiting times and increased consultation times for them, they evaluated this positively:

*'Now the consult time is increased, I feel more relaxed during the appointment. This is a change I hope will remain.'* (ID3)

Patients reported that regular care for chronic conditions, such as diabetes mellitus or pulmonary diseases, could be postponed or withheld by the GP during the COVID-19 pandemic, with some experiencing this as a negative change that had increased their stress

*'There will be situations in which health care is postponed and, as a consequence, lead to fatal situations, maybe.'* (ID4)

Responses indicated that some practices offered specific office hours for care related to COVID-19 infection, with this sometimes provided at different locations and by practitioners other than their GP. This led to a decreased quality of relationship with their own GP, and with that, to a decrease in continuity of in-person care:

*'This GP doesn’t know me. The intention of a GP should be that you know your patients.'* (ID2)

Regarding the use of personal protective equipment, patients found that this made it harder to understand the GP. Using such equipment also promoted a feeling of detachment towards the GP that caused some patients to postpone their care needs:

*'The GP is sitting there, suited and booted, wearing gloves and a facemask. This leads to a totally different conversation.'* (ID9)

Patients negatively viewed the change in ability to bring someone with them when seeing the GP, stating that this produced dissatisfaction and made it harder to remember everything the GP said:

*'Normally I would say to my husband "come along with me, so you can listen to what the doctor says as well". That is not allowed now.'* (ID23)

## Discussion

### Summary

A qualitative study was conducted to gain insights into the experiences of patients regarding GP care during the COVID-19 pandemic in The Netherlands.. After interviewing 13 male and 15 female patients (aged 27–91 years), thematic analysis identified accessibility and continuity of GP care as the main themes and the associated positive and negative consequences as sub-themes. Changes viewed as positive and valued by patients included the use of remote care for low complexity problems, the extra time scheduled for appointments, and the quieter practice. However, patients also experienced reductions in both the accessibility to, and continuity of, GP care. This included feeling unwelcome, chronic care being postponed by the GP, and seeing unfamiliar doctors owing to care being segregated.

### Strengths and limitations

This study benefitted from the inclusion of a heterogenous group of patients with and without past COVID-19 infection from regions with high and low prevalence of COVID-19. The use of both purposive sampling and assessment of saturation ensured that as many relevant experiences and opinions could be included as possible across a broad patient group. Despite these efforts, however, the authors cannot be certain that all patient experiences were included. Additionally, this study took place within the context of the Dutch healthcare system and COVID-19 containment measures. Readers should consider this context while interpreting the results.

### Comparison with existing literature

Patients in this study valued a quieter general practice that could schedule more time for consultations per patient. They reported experiencing improved quality of care from their GP because they could take their time when discussing health problems. Previous research has also shown that allowing more time for patient consultations leads to improved quality of care, higher patient satisfaction, and fewer referrals to secondary care.^19,20^

Patients experienced direct consequences in doctor–patient communication owing to the containment measures, such as the need for protective equipment and the restrictions placed on bringing a second person to a consultation. The use of personal protective equipment generated some negative experiences. It caused not only difficulties in understanding what the GP had said but also feelings of detachment towards the GP. This equipment therefore affected both verbal and non-verbal communication. A systematic review of doctor–patient communication showed that an association existed between favourable health outcomes and optimised verbal and non-verbal communication in primary care.^21^ Patients in the study also experienced impaired communication when they could not bring a relative to their consultation during the COVID-19 pandemic, stating that this made it harder to remember what their GP had said. Previous research has shown that allowing relatives to accompany patients can be beneficial, helping not only with information exchange between GP and patient, but also with healthcare coordination.^22,23^ Denying patients the opportunity to bring a relative to the consultation could lead to decreased continuity of care.

Compromising the core values of primary care can produce negative health consequences, potentially decreasing population health and even increasing mortality rates.^11–24,24^ Patients in this study reported that several measures taken during the COVID-19 pandemic, such as postponing GP care or only providing infection-related care during specific office hours or at specific locations, often with different healthcare providers, led to diminished feelings of personal contact with the GP and decreased continuity of care. This corresponds with the experiences of GPs during the first wave of the pandemic, who reported that it had profoundly affected the core values of GP care and impeded the continuity of routine care.^25^ Earlier research has also shown that most patients think a personal relationship with their GP is important and want to see the same GP on each visit.^26,27^ Continuity of care, which is a core primary care value, enhances therapy compliance and preventive care.^11,13^ Any decrease in continuity of care with the GP should be viewed with concern because it has been associated with increased specialist health care and out-of-hours service use and increased mortality.^13,24,28,29^ For example, when patients with chronic conditions (for example, diabetes) receive this lower standard of care, it can increase hospitalisation and mortality rates.^30^ Optimal continuity of care requires an adequate personal relationship between a patient and GP,^28,31^ and policies that diminish a patient’s sense of contact with their GP represent a major threat to care provision.

The results of the study are in line with previous published qualitative and quantitative studies, which reported that accessibility of primary care was impaired during the COVID-19 pandemic, in particular for specific patient groups, such as older people and those with a low educational level.^32–36^ On the other hand, it has also been shown that remote care could be an effective alternative for regular consultations and could therefore improve accessibility of primary care.^37^ However, the loss of non-verbal communication and impaired relationship between patients and GP were mentioned before as key disadvantages of remote care during the COVID-19 pandemic.^34,38^ This is comparable with the findings in the present study in which it was shown that remote care can lower the threshold for contacting the GP for low complexity problems, but could also lead to a less personal feeling with the GP. In addition, previous research showed that patients and GPs have different perceptions of advantages and disadvantages of remote care during the COVID-19 pandemic. Whereas GPs thought that short remote care contacts were more effective, patients in contrast had the feeling that they could not address everything they wanted to discuss.^38^ This emphasises the importance of gaining insights in the patient perspective, which was the focus of this study.

### Implications for research and practice

This study showed that the COVID-19 pandemic could have threatened the core values of accessibility to, and continuity of, primary care from a patient perspective. However, several organisational changes to primary care diminished the severity of impact on these core values. The challenge must now be to ensure that the carefully drafted core values of primary care continue to be observed during periods of intense pressure on healthcare systems. Further research by the group will consider the experiences of GPs regarding primary care provision during the COVID-19 pandemic in The Netherlands.. However, future quantitative research could also focus on the consequences of the COVID-19 pandemic for non-COVID-19 care, including referrals to secondary care, out-of-hours GP service use, and mortality, which could have been affected by impediments to care accessibility and continuity. It would also be interesting to compare the experiences of patients in the current study with those of patients in countries with different healthcare systems.

Promoting the positive changes experienced by patients while simultaneously minimising any negative effects will be key to improving primary care provision not only in this and future pandemics, but also when implementing similar changes to care provision in primary care. Indeed, routine primary care in the future could be improved by implementing some of the positive changes brought about by the COVID-19 pandemic, such as using remote care for low complexity problems and affording patients more time for face-to-face consultations. When we asked patients about their experiences of primary care during the COVID-19 pandemic, their answers often included concerns over the core values of primary care. This simple finding emphasises the importance of preserving these core values during periods of intense stress on primary care.
